# Strengthening Public Breeding Pipelines by Emphasizing Quantitative Genetics Principles and Open Source Data Management

**DOI:** 10.3389/fpls.2021.681624

**Published:** 2021-07-13

**Authors:** Giovanny Covarrubias-Pazaran, Johannes W. R. Martini, Michael Quinn, Gary Atlin

**Affiliations:** ^1^Excellence in Breeding Platform, Consultative Group for International Agricultural Research, Texcoco, Mexico; ^2^Genetic Resources Program, International Maize and Wheat Improvement Center, Texcoco, Mexico; ^3^Bill and Melinda Gates Foundation, Seattle, WA, United States

**Keywords:** breeding scheme optimization, quantitative genetics and breeding, genomic selection, open source, developing and transition countries

## Introduction

The strategic goals of the “Consultative Group on International Agricultural Research” (CGIAR), which serves small-scale agricultural producers in the developing world, include the increase of nutrition and food security, the reduction of poverty, and the reduction of the “environmental footprint” of agricultural production systems (https://www.cgiar.org/how-we-work/strategy/). For each of these goals, progress can be made by breeding new crop varieties with increased productivity, stress resilience, nutritional value, and reduced requirement for fertilizer or agrochemicals.

Despite the great success of CGIAR breeding in the last decades, we posit that quantitative genetics principles must be more strongly emphasized in breeding strategies to keep pace with the accelerated demand and with changes in production conditions resulting in a growing demand for food, climate change and newly introduced breeding objectives -such as nutritional quality.

Traditionally, molecular breeding approaches focused on the identification of major genes, often for disease resistance, and the introgression of these alleles into elite material. This has been a fruitful strategy to prevent or mitigate production losses since disease resistances are essential traits for most target populations of environments (TPEs). However, the focus on major genes for disease resistances may also have slowed down genetic gain for yield in some programs. We advocate the redesign of breeding pipelines with a stronger orientation on quantitative genetics principles, optimizing the components of the “breeder's equation” to deliver a high selection response for quantitative traits like yield. Moreover, to improve the basis on which selection decisions are made, we propose an open-source breeding approach in which individual public and private institutions collaborate, align their activities, and share data to enhance efficiency for all participants.

We will briefly present the breeder's equation and highlight the terms that can be manipulated to increase genetic gain per time and per dollar invested. We will also present some guidelines recommended by the Excellence in Breeding (EiB) platform to optimize the selection response in a classical breeding scheme. We then discuss how genome-assisted prediction methods (genomic selection, GS) can be used for further optimization.

## The Breeder's Equation

In its simplest form, the breeder's equation for one trait, or even a composite of traits integrated in a selection index, states that the ***genetic gain***per unit of time, expressed as the difference of the means of the (additive) genetic values before and after selection (Δ*μ*) divided by time *t*, is given by:

$$\frac{\Delta \mu }{t}=\frac{\left(h^{2}\;i\;\sigma_{p}\right)}{t}$$

Here, *h*^2^ is the narrow sense *heritability*, *i* is the standardized selection differential, that is the *difference in phenotypic standard deviations* between the mean of the selected fraction and the mean of the initial population, and *σ*_*p*_ is the *phenotypic standard deviation* of the population before selection (modified from Lynch and Walsh, 1998). The cycle length t in years describes the time needed for one breeding “cycle” including recombination, evaluation, and selection of parents for the new set of crosses. The breeder's equation highlights the parameters that can be optimized to increase genetic gain per unit of time. We can increase the accuracy of our selection, *h*^2^ for instance, by improving trial quality or increasing replication. We can increase the selection intensity *i* by selecting fewer individuals, or from a greater number of candidates (or both). Finally, we could reduce the cycle time *t* by shortening the time from cross to evaluation and to crosses of selected progeny (rapid generation advance).

## Recommendations for the Design of Progressive Breeding Pipelines

The Excellence in Breeding (EiB) platform (https://excellenceinbreeding.org/) provides guidance to the CGIAR system and its national partners on the successful implementation of genomic prediction methods. EiB has proposed, as a first step, to optimize classical programs by addressing resource allocation in the light of the breeder's equation, which may sometimes require a radical redesign of the pipeline. The routine use of genomic selection to select parents is then implemented in a second iteration. EiB has modeled many of the breeding pipelines of CGIAR centers in detail and has evaluated a range of approaches to crossing, evaluation, and selection decisions in simulations. Some general recommendations are summarized below:

1) Formalize the breeding objective by defining market segments and corresponding product profiles describing the “ideal” product.

Point (1) guarantees that we clearly define in which direction we would like to breed. Moreover, market intelligence from a wide range of sources can be brought to bear on variety design (Cobb et al., 2019). We do not advocate for a particular methodology but emphasize the importance of investing resources in de- and refining the breeding goal. Client and market intelligence can be assembled from participatory plant breeding approaches (Witcombe et al., 1996; Ashby, 2009; Ragot et al., 2018) for subsistence-oriented systems, but product design for market-oriented cropping systems requires formal engagement with farmers, processors, and marketers to ensure that breeding objectives result in products that are both producible and marketable.

2) Form the crossing blocks out of small elite populations of 20–30^1^ parents (avoid closely related individuals) and keep the crossing block as a mostly closed system. Use diversity measures and the variance of the traits defined in the product profile to monitor the diversity in the population over time.

Point (2) allows concentration on the “most elite” material (i.e., material with high breeding or genetic value) for our breeding objectives, which increases selection intensity (*i*). Moreover, a smaller effective population size avoids unnecessary crossing and testing, which saves resources. Experimental populations, theory and simulations show that a small number of elite individuals contain enough variance to avoid genetic bottlenecks in short and medium-term breeding time-horizons (Moose et al., 2004; Gaynor et al., 2017). This recommendation is linked to the breeder's equation by effectively managing the genetic variance and optimizing selection intensity.

3) The rate of new “diversity” injected into the pipeline each cycle should be low rather than high, which means parents of a cycle should be mainly chosen from the progeny of the previous cycle (recurrent selection strategy). New diversity (e.g., alleles conferring disease resistance) should be mainly injected in the form of donors of elite background with high-value haplotypes that do not currently exist in the population. This diversity must be carefully introduced to minimize linkage drag associated with new resistance alleles.

The restriction of the input of new diversity in point (3) is critical to the success of methods such as pedigree BLUP (Best Linear Unbiased Predictor) or genomic BLUP to improve the accuracy of selection of parents for the subsequent cycle. A certain degree of relatedness is required for these methods to be accurate. In addition, introgressing too many new parents can reduce the accuracy of quantitative genetics methods (Lynch and Walsh, 1998; Walsh and Lynch, 2018). When a recurrent selection strategy is used properly, almost any introgression would be a step backwards in terms of general performance and breeding value, and should only be used for special trait introgression or if genetic variance has been exhausted (Allier et al., 2020). This recommendation is linked to effectively managing the genetic variance in breeder's equation.

4) Formalize the crossing, evaluation, and selection decisions as variables in a process that is comprised of different stages (e.g., crossing blocks, nursery, early testing, late testing, etc.).

The formalization described in point (4) is required to apply selection criteria consistently and to characterize the breeding scheme more easily for simulations (point 5) and continuous improvement processes.

5) Changes in crossing, evaluation or selection procedures and resource allocations should be supported by simulations or experiments measuring the effect of the change on genetic gain while considering other influencing parameters, including costs.

It is critical that all the steps and processes used in the breeding pipeline be accurately costed, permitting simulation and modeling to be used to allocate resources to maximize the rate of genetic gain delivered per year and per dollar spent.

6) Use and document selection indices or independent culling to formalize the selection decisions when breeding for several quantitative traits simultaneously. The goal should be to make parent selection as objective and “data-driven” as possible, such that anyone having the underlying data can understand how the selection decision was made. All traits which are included in the selection decision should also be formally included in the recorded data and in the description of the selection criteria.

Selection indices allow application of selection criteria more consistently, can increase the selection intensity for several traits simultaneously and make use of genetic correlations between traits, if they are approximately known (Lynch and Walsh, 1998).

7) Use a data management and analytical system as a high priority to enable the analytical pipelines.

Adoption of analytical methods such as state of the art experimental design, spatial modeling to increase accuracy, and use of BLUP are all critical to acceleration of genetic gains. Organized, digitized data collection and storage and querying systems linking phenotypic, pedigree, and genotypic data are required to provide predictions routinely and rapidly. This recommendation is linked to all terms of the breeder's equation since better data management and analytics lead to more accurate selections, better management of diversity and in general to more accurate decisions.

8) According to the number of plots available and the breeding time-horizon, optimize the number of crosses and progeny per cross to maximize variation among and within families that can be selected.

The trade-off between allocating resources between number of families and family size will depend on factors like the number of traits included in the product profile, their genetic correlations and the time that we expect our breeding program to operate (longer periods benefit of putting more resources in the number of families and shorter breeding periods benefit of putting more resources in bigger families). We recommend the use of simulations to approach this question. This recommendation is linked to the breeder's equation by optimizing the selection intensity.

9) Parents for recycling should be selected from the first one or two testing stages of phenotyping yield (early recycling) to reduce cycle length. Also, breeders should avoid using the same parent repeatedly for several years in new crosses, which substantially lengthens the breeding cycle. Indeed, with emphasis on a short cycle time, selected progeny from a parent should always be preferred to the parent itself.

Shortening the breeding cycle while maintaining confidence in the selection of parents will often require reallocation of resources to improve data quality and quantity of the first and second testing stages of phenotyping.

10) Multiplication time (e.g., line generation, clonal propagation) should be reduced to the minimum possible (aiming for an overall cycle time as short as biology allows. For example, in seed crops that might be 2–3 years), leveraging new methodologies such as speed breeding, semi-autotrophic hydroponics, among others.

A successful example of renewing a traditional breeding pipeline at the International Rice Research Institute (IRRI) has been described at by Collard et al. (2019).

An overview, as well as a more detailed description of the different simulations supporting the recommendations above, can be found in the toolbox of EiB (https://excellenceinbreeding.org/toolbox). Once an aggressive classical breeding program with most of the features described above has been implemented, the adoption of genome-assisted prediction methods is recommended for parent selection. Implementation may follow the approach suggested below.

## Incorporation of Genomic Selection in the Breeding Strategy

Much plant breeding literature on genomic selection (GS) focuses on predictive ability, especially the prediction of the performance of a selection candidate in the absence of any phenotypic data. Predicting the commercial performance of material that has not been phenotyped, which would mean that we substitute experiments with predictions, is an important application of GS, but it is not necessarily the most impactful one, especially not for small programs. The most important application of GS is the inference of the individual's genomic estimated breeding values (GEBV) from the phenotypes of its available relatives, for the purpose of selecting parents of the next cycle. In the context of population improvement, with the objective of maximizing genetic gain per year, we are not primarily interested in the phenotype of a selection candidate itself, but rather would like to know which candidates we should select as parents of new crosses to achieve the highest improvement in the new generation. The breeding value aims at capturing the improvement of the new generation when randomly crossing the line under consideration with other lines of the population (Mrode, 2014).

The first simple step in applying GS is therefore increasing accuracy by the use of the GEBV as the selection criterion, instead of EBV or phenotypes in isolation. This application can be incorporated into any breeding pipeline, usually at the agronomic testing stage, provided that genotypic data is available. Moreover, the resulting increase in accuracy can also give more freedom to reduce cycle time *t*, for instance by allowing parents to be selected from the first stage of agronomic testing (see point 9 above) due to the increased accuracy of surrogates of trait genetic merit compared to pure phenotypic information.

A second step could be the use of GS for sparse phenotyping. Sparse phenotyping means that not each genotype is tested in each environment, but some genotypes are tested at only a subset of locations. Such an approach can increase the accuracy of the estimation of genetic values by sampling from more environments, which again reduces the error resulting from genotype-by-environment (GxE) interaction. Moreover, sparse phenotyping can be used to increase the number of candidates tested which increases selection intensity. Both, increasing the number of environments and increasing the number of tested candidates can be approached by sparse testing subjected to fixed costs. GS helps to keep the data quality when reducing the data points and models including genotype-by-environment (GxE) effects can be of additional advantage (Jarquin et al., 2020).

A third application of GS is to recycle selection candidates as early as possible (e.g. nursery stage) based on their GEBVs, or genomic estimated genetic value (GEGV; additive plus non-additive effects). The training population should be formed by phenotypes generated from related candidates from the same program (not exotic diversity panels) phenotyped in previous seasons.

The fourth step in applying GS is to use genomic marker information to predict the crossing process, e.g., not only the expected performance of genotypes coming from a certain cross, but also the variability within a family of siblings. This can be used to optimize family sizes for different crosses, and to use predicted within family variance to maximize long-term gain.

For these points see for instance Cobb et al. (2019), Clark et al. (2013), Lehermeier et al. (2017), Gorjanc and Hickey (2018) and Henryon et al. (2019), Werner et al. (2020). Any of these steps can be incorporated independently, but the order proposed reflects an increasing level of complexity of the related logistics, and therefore may lead to a more successful implementation of GS.

## Open Source Breeding

**Figure 1:**
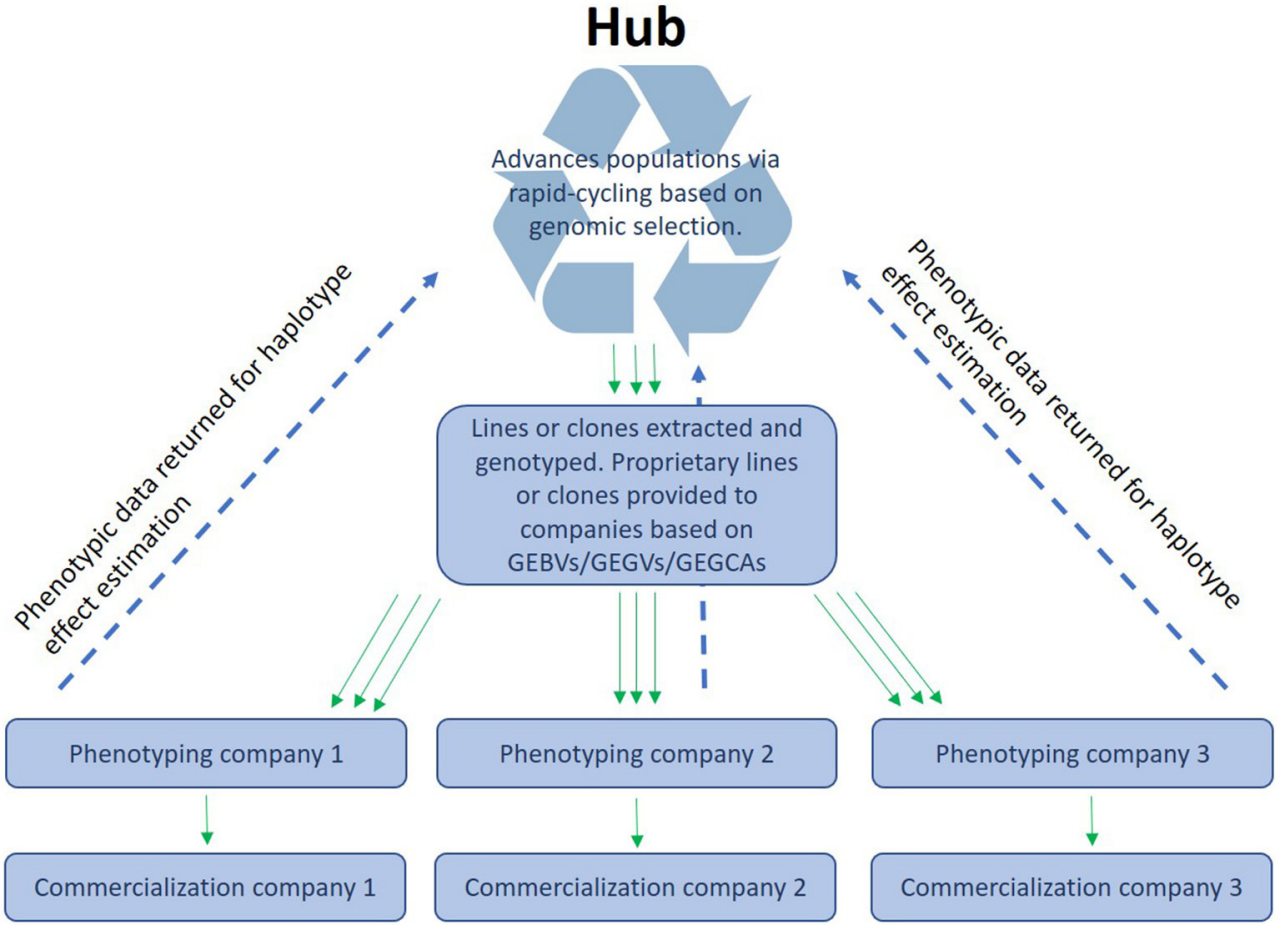
Organization of open-source breeding: A hub receives the data (phenotypes and genotypes) from different programs or companies and makes the data available as training sets to enable the different ways of using genomic prediction (see main text).

CGIAR centers together with NARs breeding centers form networks that phenotype and disseminate breeding materials that primarily originate from CGIAR centers. We envision an “open-source” breeding model that combines resources from different public and/or private partners for the benefit of all participants (intellectual property questions would need to be addressed to make a participation attractive for private partners). GS would permit the pooling of experimental data from different institutions that work within the same TPE. This would enable a better coverage of the TPE through a stronger testing network that shares (highly) related material. This way, CG centers, NARs and local companies could “borrow strength” from each other by sharing data on a central platform (Atlin and Jannink, 2010). A similar approach is currently used in dairy breeding, where the data are centrally processed and managed. In the context of public plant breeding, this would mean that the data from participating programs is jointly used to generate a stronger, more accurate prediction model than any single program could generate independently. A hub could then manage a source population and deliver lines or clones to local partners who could utilize the lines in a product development pipeline and give the experimental results back to the central data management unit (see Figure 1). Moreover, they could also use the lines as parents in their own pipelines. No CGIAR breeding networks have yet been formally constituted as open-source GS networks, but several have begun generating GEBVs for all new selection candidates and are therefore ready to implement the model with their national partners. The open-source GS network model has many advantages, including allowing breeding programs serving small-scale producers in the developing world to make selections and advance populations even when trials are lost to biotic or abiotic stress, or when disruptions such as a human pandemic hamper or prevent field testing, as happened in many breeding programs in 2020–2021. The open-source GS model will also permit highly efficient, two-stage rapid-cycle recurrent GS methods (Gaynor et al., 2017) that can reduce the breeding cycle to the biological limit imposed by the juvenility period of the species (time interval needed to move from seed to seed in seed crops may be 1 year or less but in tree species may be a couple of years) to be applied in the service of small-scale producers in Africa.

## Conclusion

An efficient implementation of genomic prediction methods in CGIAR-NARs breeding programs (and maybe other publicly funded programs) depends on forming structured programs that follow certain design rules. Such programs must be outcome-oriented, with well-defined targets expressed in formal product profiles that guide selection decisions. We suggest that the first step in this process is to implement a classical breeding pipeline optimized based on quantitative genetics principles (reducing cycle time to the biological limit while increasing the accuracy of early testing and managing the genetic diversity at the proper program size). From there, the adoption of GS methods will be a natural extension guided by the breeder's equation. A first step would then be the use of GEBVs as selection criteria instead of phenotypic data in isolation. The breeding populations should be (almost) closed, using a relatively small number of elite parents. In the next steps, GS should be used to reduce evaluation costs while increasing the coverage of the TPE using sparse testing supported by marker data. Moreover, GS should be used to reduce the breeding cycles down to 1 year in a stepwise fashion for most crops if the phenotyping and selection methods are up to the challenge (data for all traits and use of indices is a pre-requisite for the most extreme use of GS). Simultaneously, it should be explored how “open source” breeding structures could be implemented in CGIAR-NARs networks, allowing small breeding programs to borrow strength from each other by incorporating the data generated by other programs working in the same crop but different regions with highly related material.

## Author Contributions

GC-P, JM, MQ, and GA wrote the manuscript and conceived the ideas of the manuscript. All authors contributed to the article and approved the submitted version.

## Conflict of Interest

The authors declare that the research was conducted in the absence of any commercial or financial relationships that could be construed as a potential conflict of interest.

## Footnotes

^1^The number of elite parents is suggested for 30-year breeding time horizon of a classical program that takes between 3–5 years to recycle parents. In addition, the number of elite parents in the crossing block must be increased when adopting an aggressive GS scheme (recycling F1s) because the number of effective cohorts decreases drastically.
